# Bilirubin Levels Are Negatively Correlated with Adiposity in Obese Men and Women, and Its Catabolized Product, Urobilin, Is Positively Associated with Insulin Resistance

**DOI:** 10.3390/antiox12010170

**Published:** 2023-01-11

**Authors:** Zachary A. Kipp, Mei Xu, Evelyn A. Bates, Wang-Hsin Lee, Philip A. Kern, Terry D. Hinds

**Affiliations:** 1 Department of Pharmacology and Nutritional Sciences, University of Kentucky, 760 Press Avenue, Healthy Kentucky Research Building, Lexington, KY 40508, USA; 2 Department of Internal Medicine, Division of Endocrinology, University of Kentucky, Lexington, KY 40508, USA; 3 Barnstable Brown Diabetes Center, University of Kentucky, Lexington, KY 40508, USA; 4 Markey Cancer Center, University of Kentucky, Lexington, KY 40508, USA

**Keywords:** bilirubin, urobilin, UGT1A1, HO-1, BVRA, obesity, insulin resistance, blood glucose, BMI, HOMA IR

## Abstract

Bilirubin levels in obese humans and rodents have been shown to be lower than in their lean counterparts. Some studies have proposed that the glucuronyl UGT1A1 enzyme that clears bilirubin from the blood increases in the liver with obesity. UGT1A1 clearance of bilirubin allows more conjugated bilirubin to enter the intestine, where it is catabolized into urobilin, which can be then absorbed via the hepatic portal vein. We hypothesized that when bilirubin levels are decreased, the urobilin increases in the plasma of obese humans, as compared to lean humans. To test this, we measured plasma levels of bilirubin and urobilin, body mass index (BMI), adiposity, blood glucose and insulin, and HOMA IR in a small cohort of obese and lean men and women. We found that bilirubin levels negatively correlated with BMI and adiposity in obese men and women, as compared to their lean counterparts. Contrarily, urobilin levels were positively associated with adiposity and BMI. Only obese women were found to be insulin resistant based on significantly higher HOMA IR, as compared to lean women. The urobilin levels were positively associated with HOMA IR in both groups, but women had a stronger linear correlation. These studies indicate that plasma urobilin levels are associated with obesity and its comorbidities, such as insulin resistance.

## 1. Introduction

The worldwide rates of obesity are at an all-time high, which is characterized by a body mass index (BMI, kg/m^2^) greater than 30 [1]. While excessive food intake and a sedentary lifestyle are the primary drivers of increased obesity, there has been little progress in developing effective treatments for reducing adiposity and body weight. There have been many large omics analyses, such as RNA sequencing, that have indicated a genetic component for obesity. However, some factors are not observable in these types of extensive bioinformatic analyses. In the context of obesity, how physiological responses to weight gain affect the functionality of the detoxifying cytochrome P450 or glucuronyl UGT enzymes in regulating catabolism and the transport of non-gene-related molecules is mostly unexplored.

One notable non-gene-derived molecule is bilirubin, which is a catabolized product of heme [2,3,4,5,6,7] that, for unknown reasons, has been shown to be lower in obese humans and rodents [2,3]. Bilirubin is a strong antioxidant and robust anti-inflammatory [8,9,10,11] that has been shown to reduce body weight in obese rodents via its hormonal actions on the PPARα nuclear receptor [2,3,5,6,12,13,14,15,16,17]. Some studies have demonstrated that the glucuronyl UGT1A1 enzyme that clears bilirubin from the blood increases in the liver with obesity [18,19]. The UGT1A1 glucuronosyltransferase conjugates bilirubin to lower levels (clearance) [20]. The conjugated bilirubin is excreted to bile and then to the intestine, where it is catabolized into urobilin [6]. The freshly produced urobilin can be absorbed and enter the systemic circulation via the hepatic portal vein [6]. The physiological function of urobilin has not been well studied. In humans, urobilin plasma levels are associated with a higher visceral fat area in the obese [21], heart failure [22], and all-cause mortality in diabetic patients [23].

Here, we provide intriguing data that urobilin levels are positively associated with adiposity and BMI in obese men and women and also with insulin resistance in women, which was determined using the Homeostatic Model Assessment for Insulin Resistance (HOMA IR), a measurement of insulin sensitivity [24]. We demonstrate that plasma urobilin and bilirubin levels are inversely correlated. Our data indicate that urobilin is a factor in obese people that may be linked with worsened obesity-associated comorbidities, such as insulin-resistant diabetes. The findings in this study help to understand better the factors that are changed in obesity and how these are related to obesity-induced comorbidities.

## 2. Materials and Methods

### 2.1. Human Subject Participation

Blood-derived phenotypic data were collected from adult participants (aged 18–80 years) donated to the Center for Muscle Biology (CMB) at the University of Kentucky under an Institutional Review Board (IRB), which was reviewed and approved (IRB protocol #46746), using the Center for Clinical and Translational Science (CCTS) grant #UL1TR001998. Samples were selected based on a BMI >30 for obese women and men (*n* = 13 total (obese women *n* = 8 and obese men *n* = 5)) and a BMI <25 for lean (*n* = 17 total (lean women *n* = 8 and lean men *n* = 9)). None of the obese subjects had diabetes, hypertension, heart disease, or other significant complications. All healthy volunteers did not report any known evidence of cardiovascular, metabolic, or neuromuscular disease and were non-smokers. All data and blood samples were collected during the same visit after at least an 8 h fast. Height and weight were collected using the same stadiometer and scale, whereas body mass index (BMI) was calculated as kg/m^2^. Body composition was determined using a Lunar Prodigy (GE Lunar Inc., Madison, WI) dual-energy X-ray absorptiometry (DEXA) bone densitometer and analyzed with GE Lunar software version 10.0 software for bone mineral content (BMC), bone mineral density (BMD), fat-free mass (FFM), mineral-free lean mass (MFL), fat mass, and fat percent. The patients fasted, and blood was collected (20–30 mL) by venipuncture. Fasting glucose and insulin levels were measured by a YSI 2900 Biochemistry Analyzer and an Insulin Chemiluminescence ELISA (ALPCO), respectively. HOMA IR was calculated by the following formula: HOMA-IR = (fasting glucose × fasting insulin)/22.5 [24]. The additional blood that was stored at the CMB was used for additional measures, such as bilirubin and urobilin measures, as described below. The data in Table 1 and Table 2 are displayed in mean ± standard deviation.

### 2.2. Plasma Bilirubin Measurements

Total bilirubin was measured by using a colorimetric method according to the manufacturer’s kit booklet (Cayman Chemical, Ann Arbor, MI, USA). Briefly, after adding 100 µL of bilirubin assay catalyst into each well of 96-well plates, 50 µL of plasma was added into designated wells, followed by 10 min of incubation at room temperature. After another incubation of 15 min following the addition of 25 µL of freshly prepared reagent mix or background mix, 75 µL of total bilirubin probe was added into each well, followed by a third incubation period for 5 min. Then, the absorbance was read at 600 nm. The total bilirubin concentration of each sample was calculated based on the standard curve.

### 2.3. Plasma Urobilin Measurements

Plasma urobilin was measured by spectrophotometry, which was performed as described before [25,26,27]. Urobilin hydrochloride (Frontier Specialty Chemicals, Logan, UT, USA) was dissolved in DMSO to make the urobilin standard curve using 0~125 µM urobilin. DMSO/saline was used as the blank of standard/plasma. All blanks, standards, and samples were attained using the same extraction method to form the oxidation products of urobilin-zinc complexes. Briefly, 10 µL of standards or plasma samples were added to 60 µL of 54 mM zinc acetate (in DMSO) in each tube, followed by adding 12 µL of 25 mM iodine (dissolved in 120 mM potassium iodine). After vigorously mixing for 30 s, 5 µL of 82 mM cysteine HCL was added to each sample, followed by another vortex. Then, they were centrifuged at 5000× *g* for 3 min. First, supernatants were collected into the newly designated tubes. Each pellet underwent a repeated extraction. Two supernatants were combined. A total of 50 µL of 1 M HCL was added to the blanks to eliminate the background fluorescence. The liquid extracted from each sample was added to a 96-well plate, and then absorbance was read at 508 nm. Each sample was performed in duplicate. The urobilin concentration of the samples was calculated using the linear regression equation of the standard curve.

### 2.4. Statistical Analysis

The data were graphed and analyzed using Prism 9 (GraphPad Software, San Diego, CA, USA), and an analysis of variance and Tukey’s post hoc test were used to compare the groups’ means. A two-tailed unpaired *t*-test was used to determine statistical significance when comparing the two groups. To determine correlation, a linear regression line was fitted to the data, and the linear equation and R^2^ value were calculated for each comparison. Finally, *p*-values of 0.05 or smaller were considered statistically significant.

## 3. Results

### 3.1. Bilirubin and Urobilin Levels in Obese and Lean Men and Women

#### Phenotyping of Obese and Lean Men and Women

To determine the relationship between bilirubin and urobilin in obese and lean men and women, body weight, BMI, total fat, and body fat percentage were quantitated, and blood was drawn for the plasma analysis of the levels of total bilirubin, urobilin, glucose, and insulin (Table 1 and Table 2). The glucose and insulin levels were used for quantifying the HOMA IR.

The plasma measurements show that bilirubin levels were lower in obese men and women but not significantly. The urobilin levels were significantly (*p* = 0.0262) higher in the obese subjects, as compared to the lean. However, this was only seen in obese females (*p* = 0.0278) and not in obese males. One factor that might regulate these differences is that the obese women were insulin resistant, as indicated by a significantly higher insulin level (*p* = 0.0127) and HOMA IR (*p* = 0.0160). Next, to determine the correlation of urobilin or bilirubin with these metabolic dysfunctions, we performed Pearson’s coefficients to calculate their relationships.

### 3.2. The Relationship between Urobilin and Bilirubin Levels

**Figure 1:**
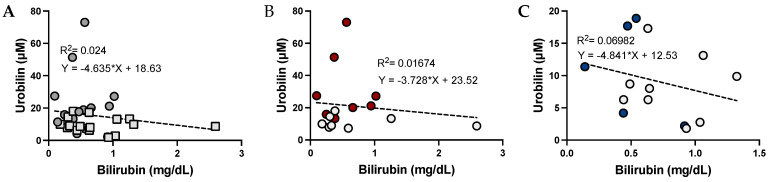
**The relationship between urobilin and total bilirubin levels in women and men.** Pearson’s coefficient comparisons in all samples from men and women to determine the slope of the line and the relationship between urobilin and bilirubin in lean participants of both sexes (**A**), only women (**B**) or only men (**C**). The data were plotted as dot plots, linear regression was calculated, and the slope of the line was used to determine the relationship and the robustness of the data. Light gray squares are for lean women and men combined, and dark gray circles are for obese men and women combined. Red circles are for obese women, blue circles are for obese men, and gray circles are for their lean counterparts.

To determine whether urobilin and bilirubin levels are interrelated, we performed a Pearson’s coefficient graph with a line to fit the data (Figure 1). The data show that urobilin is negatively associated with bilirubin levels in women and men.

### 3.3. Urobilin and Its Correlation with Metabolic Dysfunction in Humans

**Figure 2:**
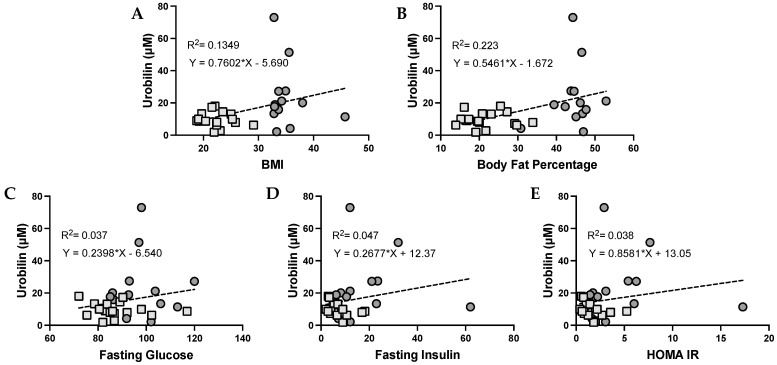
**Urobilin levels and correlations with metabolic phenotypes in women and men**. Comparisons of metabolic phenotypes in women and men with plasma urobilin levels for (**A**) BMI, (**B**) body fat percentage, (**C**) blood glucose, (**D**) blood insulin, and (**E**) HOMA IR. The data were plotted as dot plots, linear regression was calculated, and the slope of the line was used to determine the relationship and the robustness of the data. Light gray squares are for lean women and men combined, and dark gray circles are for obese men and women combined.

**Figure 3:**
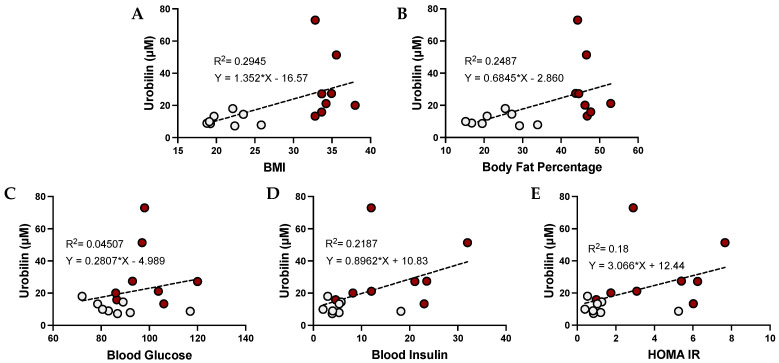
**Urobilin levels and correlations with metabolic phenotypes in women**. Comparisons of metabolic phenotypes in women with plasma urobilin levels for (**A**) BMI, (**B**) body fat percentage, (**C**) blood glucose, (**D**) blood insulin, and (**E**) HOMA IR. The data were plotted as dot plots, linear regression was calculated, and the slope of the line was used to determine the relationship and the robustness of the data. Red circles are for obese women, and gray circles are for their lean counterparts.

**Figure 4:**
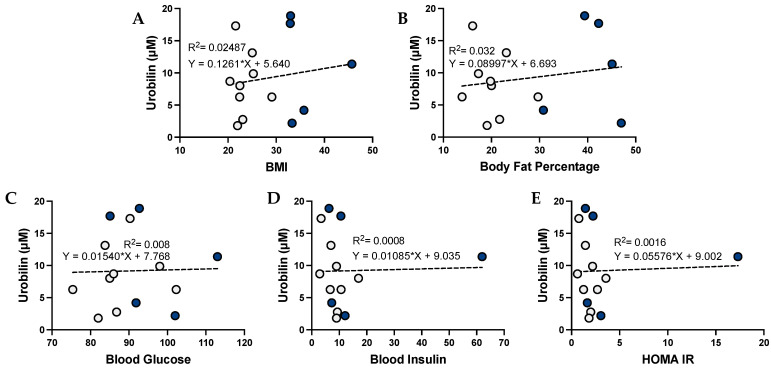
**Urobilin levels and correlations with metabolic phenotypes in men**. Comparisons of metabolic phenotypes in men with plasma urobilin levels for (**A**) BMI, (**B**) body fat percentage, (**C**) blood glucose, (**D**) blood insulin, and (**E**) HOMA IR. The data were plotted as dot plots, linear regression was calculated, and the slope of the line was used to determine the relationship and the robustness of the data. Blue circles are for obese men, and gray circles are for their lean counterparts.

The level of urobilin in the plasma has been previously associated with adiposity. We found that urobilin was positively correlated with BMI (*p* = 0.0459), body fat percentage (*p* = 0.0084), plasma glucose, plasma insulin, and HOMA IR (Figure 2). We then wanted to analyze whether this correlation was sex specific. We found that urobilin was positively correlated with higher BMI, body fat percentage, plasma insulin, and HOMA IR in females (Figure 3). In men, urobilin levels were positively associated with these but weakly with R^2^ below 0.03 (Figure 4).

### 3.4. Bilirubin and Its Correlation with Metabolic Dysfunction in Humans

**Figure 5:**
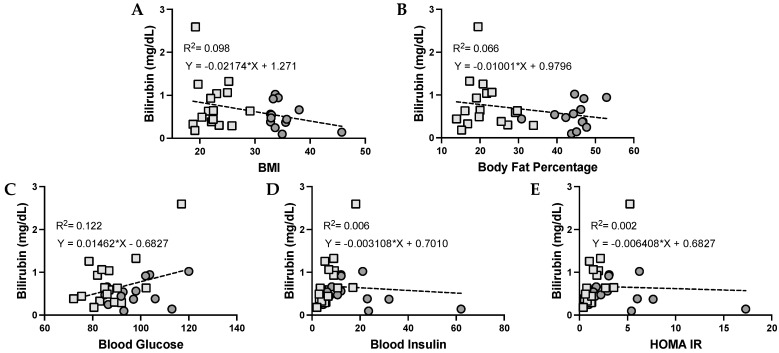
**Bilirubin levels and correlations with metabolic phenotypes in women and men**. Comparisons of metabolic phenotypes in women and men with plasma bilirubin levels for (**A**) BMI, (**B**) body fat percentage, (**C**) blood glucose, (**D**) blood insulin, and (**E**) HOMA IR. The data were plotted as dot plots, linear regression was calculated, and the slope of the line was used to determine the relationship and the robustness of the data. Light gray squares are for lean women and men combined, and dark gray circles are for obese men and women combined.

**Figure 6:**
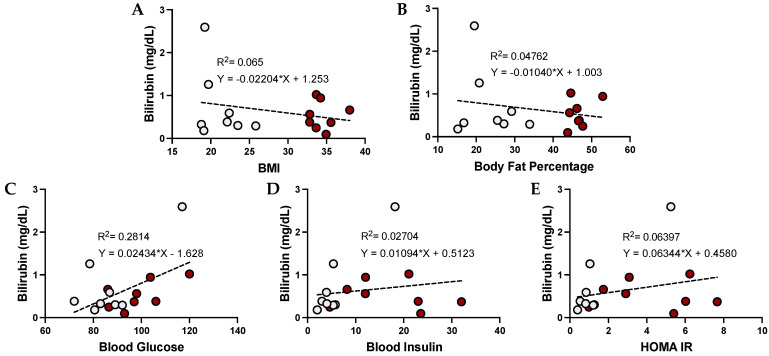
**Bilirubin levels and correlations with metabolic phenotypes in women**. Comparisons of metabolic phenotypes in women with plasma bilirubin levels for (**A**) BMI, (**B**) body fat percentage, (**C**) blood glucose, (**D**) blood insulin, and (**E**) HOMA IR. The data were plotted as dot plots, linear regression was calculated, and the slope of the line was used to determine the relationship and the robustness of the data. Red circles are for obese women, and gray circles are for their lean counterparts.

**Figure 7:**
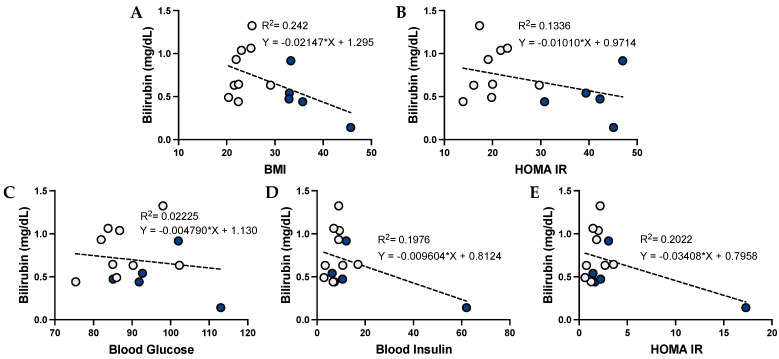
**Bilirubin levels and correlations with metabolic phenotypes in men**. Comparisons of metabolic phenotypes in men with plasma bilirubin levels for (**A**) BMI, (**B**) body fat percentage, (**C**) blood glucose, (**D**) blood insulin, and (**E**) HOMA IR. The data were plotted as dot plots, linear regression was calculated, and the slope of the line was used to determine the relationship and the robustness of the data. Blue circles are for obese men, and gray circles are for their lean counterparts.

To determine whether bilirubin might have the opposite correlations to those observed with urobilin, we compared plasma total bilirubin levels with the metabolic readouts. Plasma bilirubin levels were negatively associated with BMI (*p* = 0.0924) and body fat percentage in women and men combined (Figure 5). This association was still seen when the sexes were analyzed separately (Figure 6 and Figure 7). Interestingly, the negative association was stronger in males, with an R^2^ = 0.242 for BMI and R^2^ = 0.1336 for body fat percentage, as compared to R^2^ = 0.065 in females for BMI and R^2^ = 0.047 for body fat percentage.

### 3.5. Graphical Representation of the Data

**Figure 8:**
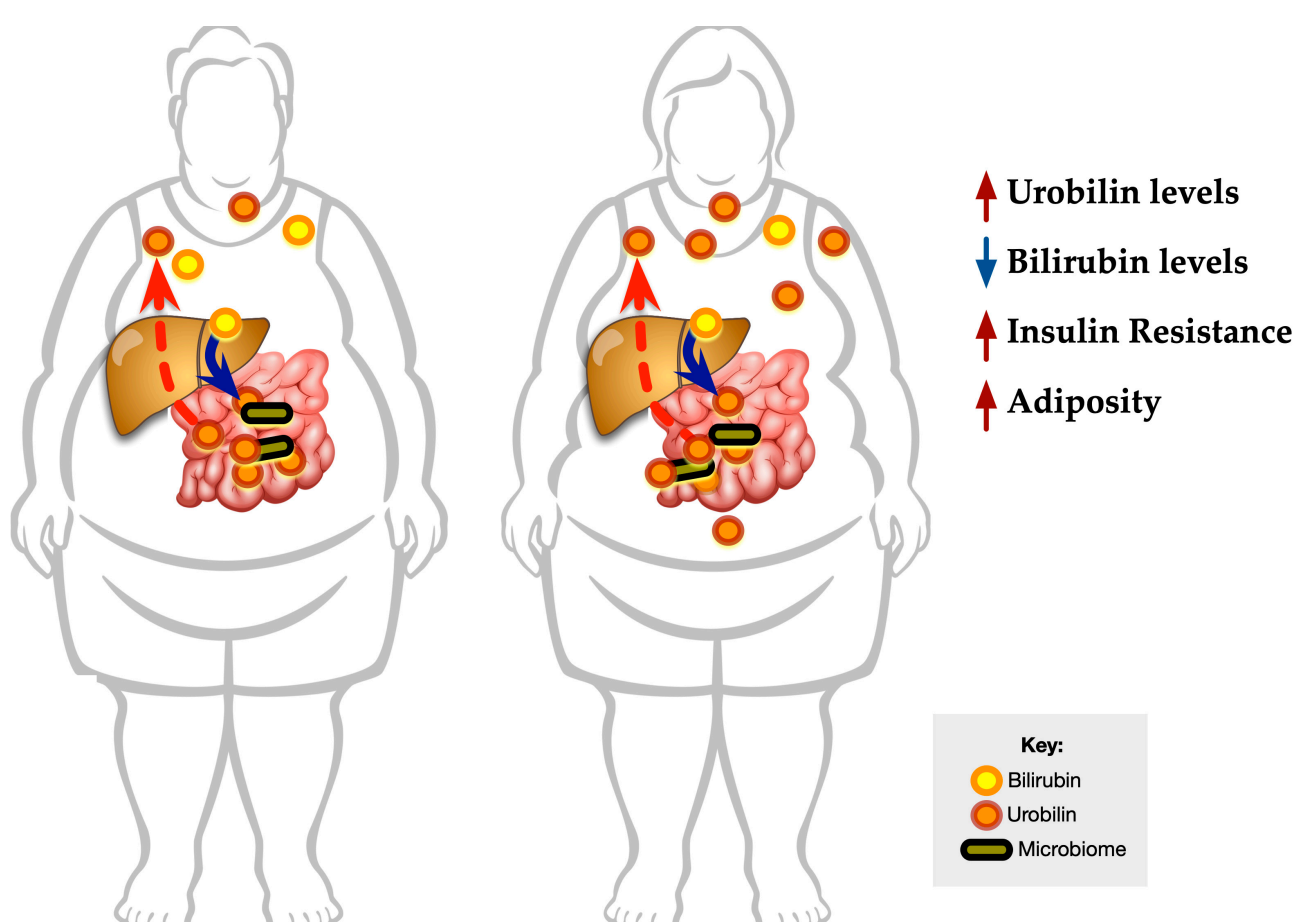
**Urobilin and bilirubin levels in obesity**. Bilirubin is conjugated in the liver and then catabolized in the intestine to form urobilin by the gut microbiome, which can be absorbed via the hepatic portal vein. The plasma levels of urobilin are positively associated with adiposity and BMI and might be a regulator of insulin resistance. Bilirubin is negatively associated with adiposity and BMI.

The data above indicate that higher plasma urobilin levels are associated with insulin resistance in the obese. The metabolic rearrangements in obesity are likely linked to lower plasma bilirubin and higher urobilin levels. Additionally, urobilin might be a factor that worsens obesity-associated comorbidities, such as insulin resistance and cardiovascular diseases (Figure 8).

## 4. Discussion

The significance of our study is in the finding that urobilin may have negative consequences pertinent to insulin resistance and may worsen obesity-related comorbidities. To our knowledge, the involvement of urobilin in insulin resistance in humans has not previously been reported. In experimental animals, studies in obese and lean mice using non-targeting mass spectrophotometry found that urobilin was higher in the liver and colon of the obese [28,29], and urobilin was the most associated cecal metabolite for acute myocardial ischemia [29]. Whether nutrients from the diet or other factors increase urobilin levels are unknown. Earlier studies in the late 1990s on bilirubin showed that plasma levels are typically reduced in obese humans, which influenced numerous studies in humans and rodents comparing the levels in those with metabolic dysfunction (reviewed in [2]).

In 1997, work by Torgerson et al. first showed that plasma bilirubin levels were decreased in humans with metabolic syndrome, even though they had elevated aminotransferases (AST and ALT) [30]. We found a similar finding with bilirubin levels in our study, such as that obese men and women had a negative correlation with adiposity and body weight. The studies that demonstrated that the UGT1A1 enzyme is higher in obese mice [18,19] made us curious whether lower bilirubin levels were associated with an increase in its catabolized product, urobilin. Our data here from obese and lean women and men indicate that urobilin might be a factor that either contributes to adiposity or might worsen obesity-associated comorbidities.

The urobilin levels were likely raised due to the increased expression of the UGT1A1 glucuronosyltransferase that conjugates bilirubin [20], providing more substrates to the gut for catabolism into urobilin [6]. The UGT1A1 expression has been shown to be higher in obese mice, as compared to lean mice [18,19]. This aspect is in line with the findings that plasma bilirubin levels are lower in the obese. However, these mechanisms have yet to be proven and could be multifactorial. For instance, a study in high aerobic-capacity running rats showed that they have significantly higher plasma bilirubin levels because the enzyme that converts biliverdin to bilirubin, biliverdin reductase A (BVRA) [7], was significantly higher in the liver, and UGT1A1 was lower [31]. Some studies have focused solely on the induction of heme oxygenase-1 (HO-1) as the means of increasing plasma bilirubin levels [32]. None of the studies that have measured the heme oxygenase pathway have also analyzed the urobilin levels in the serum or intestine. Based on our findings here, this might be useful for understanding the metabolic state.

In a recent study in elite athletes, serum bilirubin levels were observed to be substantially higher [33]. However, only the bilirubin levels were quantitated and not the urobilin. They also found that the prevalence of Gilbert’s syndrome and hyperbilirubinemia in athletes was significant [33]. These observations suggest that moderately increasing plasma bilirubin predisposes a better physical performance, which is also supported by the finding that regular physical activity elevates serum bilirubin concentrations [34,35] (and was also reviewed in [36,37]). Rats that had a loss-of-function mutation in their *Ugt1a1* gene exhibited hyperbilirubinemia and were protected against hypertension and end-stage organ damage [38,39,40,41,42]. New findings have shown that bilirubin is a hormone that directly binds to the fat-burning nuclear receptor PPARα [13,15,17]. A knockout of the enzyme that generates bilirubin, BVRA, causes a bilirubin deficiency that induces oxidative stress, increases lipid accumulation, and reduces PPARα activity [12,43,44,45,46,47]. The bilirubin-PPARα interaction activates a response that lowers lipid accumulation in the liver and adipose tissues [2,3,4,5,6,13,14,15,16,17,31,48,49,50]. Hence, the therapeutic use of bilirubin has been proposed for improving obesity and metabolic dysfunction [2,3,5].

The antioxidant [8,9,10], anti-inflammatory [36], and fat-burning traits of bilirubin make it an ideal therapeutic, especially since it has been shown to also improve cardiovascular function [2,3,4,5,11,12,31,32,48,49,51,52,53]. Bilirubin nanoparticle treatments in obese mice have been shown to increase lean mass, reduce adiposity, and lower blood glucose [15]. Numerous human studies have shown that unconjugated bilirubin levels are inversely associated with non-alcoholic fatty liver disease (NAFLD) and non-alcoholic steatohepatitis (NASH) [30,54,55,56,57,58]. There has been some published work showing that unconjugated bilirubin levels could be higher in some fatty liver patients or were not causally associated with a decreased risk of NAFLD [59,60]. Another study using a diet-induced obesity fatty liver model demonstrated that increasing plasma bilirubin and bilirubin nanoparticle treatments improved NAFLD while not causing liver toxicity [14,17,50], they actually improved NAFLD and liver function by reducing the AST liver dysfunction biomarker, lowered hepatic inflammation, and decreased the percent liver fat content [14,50]. These suggest that higher bilirubin levels may promote physical and hepatic health.

The negative correlation of plasma bilirubin found in obese men and women in this study is not surprising, as this has been reported numerous times. There is plausible evidence that low bilirubin levels are deleterious and contribute to metabolic diseases [30,61,62,63,64,65,66,67,68,69]. Investigations on factors that mediate bilirubin and urobilin levels are needed to understand their interplay better. For instance, the finding that urobilin levels were not significantly higher in obese men but were in women was surprising. There could be many variables involved, such as that the men were not insulin resistant. Another could be that the gut microbiome may be different between the sexes and contain fewer bacteria that express bilirubin reductase, which is the enzyme thought to catabolize conjugated bilirubin into urobilin [6]. However, the bilirubin reductase enzyme has yet to be identified. Studies to identify bilirubin reductase and the bacteria that express it are needed. An approach being investigated is the suppression of the hepatic UGT1A1 for increasing the half-life of circulating bilirubin as a possible means of treating obesity [2].

Humans with Gilbert’s polymorphism UGT1A1*28 have a significantly reduced expression of the UGT1A1 enzyme as a result of a base pair insertion in the gene promoter (TA_7/7_ promoter gene variant) [2]. Studies have shown that patients with Gilbert’s polymorphism and higher plasma bilirubin levels have less incidence of metabolic diseases, such as obesity and type 2 diabetes mellitus (T2DM) [2,6,70]. Experimental studies in mice with the human Gilbert’s syndrome polymorphism (UGT1A1*28) were shown to have moderate hyperbilirubinemia and were protected from high-fat diet-induced hepatic steatosis (NAFLD) and insulin resistance [50]. The studies here found that plasma total bilirubin and urobilin are inversely associated, likely due to increased UGT1A1 expression, indicating that regulating its expression may benefit metabolic dysfunction.

## 5. Conclusions

A better understanding of the indications of plasma levels of bilirubin and urobilin is needed as well as what implications their levels have in various disease states. Our study here, using a small cohort of lean and obese humans, is the first to report that plasma urobilin levels are positively associated with obesity-induced comorbidities, such as insulin resistance. It should be noted that a limitation of this study is the sample size, and larger investigations are needed to determine whether urobilin is indeed a player in adiposity and insulin resistance, including pre-clinical studies to determine causality. Others have reported that urobilin levels are associated with significantly more visceral fat in obese humans [21], cardiovascular disease [22], and mortality in diabetic patients [23]. These findings indicate that larger human association studies are needed to better understand the impact that urobilin might have on obesity-induced comorbidities. There needs to be more known about the physiology and function of urobilin. Studies to identify bilirubin reductase in bacteria and those that express it are also needed. Future studies to determine mechanisms that reduce urobilin and increase bilirubin levels in the plasma could benefit those with metabolic and cardiovascular complications.

## Figures and Tables

**Table 1:** Phenotyping of obese and lean humans.

Measurement	Lean	Obese	*p*-Value
Age	31.55 ± 13.09	40.91 ± 17.11	0.632
BMI	22.47 ± 2.73	35.11 ± 3.53	0.057
Body Weight (kg)	64.60 ± 11.03	99.37 ± 12.21	0.256
Total Fat (kg)	14.73 ± 4.80	45.28 ± 7.33	0.022 *
Body Fat %	21.69 ± 5.66	44.42 ± 5.16	0.003 *
Total Bilirubin (mg/dL)	0.77 ± 0.59	0.52 ± 0.30	0.529
Urobilin (μM)	9.57 ± 4.50	23.38 ± 19.30	0.026 *
Blood Glucose	87.57 ± 10.66	98.06 ± 10.64	0.246
Blood Insulin	7.21 ± 4.66	18.06 ± 15.51	0.398
HOMA IR	1.63 ± 1.25	4.59 ± 4.37	0.398

* denotes *p* < 0.05.

**Table 2:** Phenotyping of obese and lean men and women.

Measurement	Lean Female	Obese Female	*p*-Value	Lean Male	Obese Male	*p*-Value
Age	29.76 ± 12.82	39.31 ± 12.61	0.155	33.14 ± 13.91	43.45 ± 24.23	0.4180
BMI	21.33 ± 2.54	34.46 ± 1.72	3.40 × 10^−8^ *	23.47 ± 2.62	36.14 ± 5.48	0.0045 *
Body Weight (g)	56.11 ± 7.21	93.07 ± 7.85	1.25 × 10^−7^ *	72.14 ± 7.85	109.42 ± 11.59	0.0006 *
Total Fat (g)	14.05 ± 4.97	44.68 ± 6.29	5.88 × 10^−8^ *	15.32 ± 4.86	46.24 ± 9.50	0.0008 *
Body Fat %	23.51 ± 6.50	46.61 ± 2.88	4.38 × 10^−6^ *	20.08 ± 4.57	40.92 ± 6.35	0.0005 *
Total Bilirubin (mg/dL)	0.74 ± 0.82	0.54 ± 0.33	0.527	0.79 ± 0.30	0.50 ± 0.28	0.0954
Urobilin (μM)	11.07 ± 3.79	31.21 ± 20.58	0.028 *	8.23 ± 4.85	10.86 ± 7.60	0.5108
Blood Glucose	87.38 ± 13.53	98.78 ± 11.21	0.089	87.73 ± 8.19	96.93 ± 10.81	0.1440
Blood Insulin	5.95 ± 5.11	17.06 ± 9.26	0.013 *	8.33 ± 4.20	19.64 ± 23.8	0.3493
HOMA IR	1.42 ± 1.52	4.26 ± 2.40	0.016 *	1.81 ± 0.94	5.13 ± 6.83	0.3394

* denotes *p* < 0.05.

## Data Availability

The data presented in this study are available on request from the corresponding author.
